# Detection and segmentation of morphologically complex eukaryotic cells in fluorescence microscopy images via feature pyramid fusion

**DOI:** 10.1371/journal.pcbi.1008179

**Published:** 2020-09-08

**Authors:** Nikolaus Korfhage, Markus Mühling, Stephan Ringshandl, Anke Becker, Bernd Schmeck, Bernd Freisleben

**Affiliations:** 1 Department of Mathematics and Computer Science, Philipps-Universität Marburg, Marburg, Germany; 2 LOEWE-Center for Synthetic Microbiology, Philipps-Universität Marburg, Marburg, Germany; 3 Institute for Lung Research, Universities of Gießen and Marburg Lung Center, Marburg, Germany; 4 Department of Medicine, Pulmonary and Critical Care Medicine, Philipps-Universität Marburg, Germany; Hebrew University of Jerusalem, ISRAEL

## Abstract

Detection and segmentation of macrophage cells in fluorescence microscopy images is a challenging problem, mainly due to crowded cells, variation in shapes, and morphological complexity. We present a new deep learning approach for cell detection and segmentation that incorporates previously learned nucleus features. A novel fusion of feature pyramids for nucleus detection and segmentation with feature pyramids for cell detection and segmentation is used to improve performance on a microscopic image dataset created by us and provided for public use, containing both nucleus and cell signals. Our experimental results indicate that cell detection and segmentation performance significantly benefit from the fusion of previously learned nucleus features. The proposed feature pyramid fusion architecture clearly outperforms a state-of-the-art Mask R-CNN approach for cell detection and segmentation with relative mean average precision improvements of up to 23.88% and 23.17%, respectively.

## Author summary

To analyze cell infection and changes in cell morphology in fluorescence microscopy images, proper cell detection and segmentation are required. In automated fluorescence microscopy image analysis, the separation of signals in close proximity is a challenging problem. High cell densities or cluster formations increase the probability of such situations on the cellular level. Another limitation is the detection of morphologically complex cells, such as macrophages or neurons. Their indefinite morphology causes identification issues when looking for slight variations of fixed shapes. Compared to merely segmenting cytoplasm, instance-based segmentation is a much harder task, since the assignment of a cell instance identity to every pixel of the image is required. We present a new deep learning approach for cell detection and segmentation that efficiently utilizes the nucleus channel for improving segmentation and detection performance of cells by incorporating previously learned nucleus features. This is achieved by a fusion of feature pyramids for nucleus detection and segmentation. The performance is evaluated on a microscopic image dataset, containing both nucleus and cell signals.

This is a *PLOS Computational Biology* Software paper.

## Introduction

High-throughput cell biology incorporates methods such as microscopic image analysis, gene expression microarrays, or genome-wide screening to address biological questions that are otherwise unattainable using more conventional methods [1].

Nevertheless, fluorescence microscopy is often avoided in high-throughput experiments, since the generated data is tedious to analyze by humans or too complex to analyze using available image processing tools. However, flourescence microscopy offers information about subcellular localization, supports morphological analysis, and permits investigations on the single cell level [2].

A limiting factor of automated fluorescence microscopy image analysis is the separation of signals in close proximity, regardless of whether signals originate from neighboring cells or single spots. High cell densities or cluster formation increase the probability of such situations on the cellular level [3], while high background or low spatial resolution complicate the problem on the signal level. Another limitation is the detection of morphologically complex cells, such as macrophages or neurons. Their indefinite morphology causes identification issues when looking for slight variations of fixed shapes.

The fluorescence microscopy images considered in this paper originate from high-throughput screening, with the aim of analyzing phenotypic changes and differences in bacterial infection rates of macrophages upon treatment. To analyze cell infection and changes in cell morphology, proper cell detection and segmentation are required. Upon adhesion to a surface, these cells tend to form clusters showing faint signal changes in areas with cell to cell contact. These restrictions prevent appropriate analysis with conventional microscopy analysis software.

**Fig 1:**
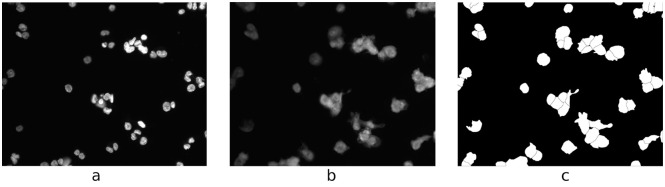
Image with ground truth segmentation. Nucleus signal (a), cytoplasm signal (b), and ground truth segmentation (c).

Compared to merely segmenting cytoplasm, instance-based segmentation is a much harder task, since the assignment of a cell instance identity to every pixel of an image is required. Fig 1 shows that the accurate separation and segmentation of clustered cells without any further information is extremely difficult, if not impossible. Even for human experts, the correct separation of individual cells is often only possible using nucleus information. Fig 1 indicates that taking the nucleus signal into account alleviates the identification of individual cells significantly.

In this paper, a deep learning approach to cell detection and segmentation based on a convolutional neural network (CNN) architecture is applied to fluorescence microscopy images containing channels for nuclei and cells. The contributions of the paper are as follows:

- We provide a novel dataset of macrophage cells for public use, including ground truth bounding boxes and segmentation masks for cell and nucleus instances.
- We utilize nucleus information in a deep learning approach for improved cell detection and segmentation. The nucleus channel is used to improve the quality of cell detection and segmentation. To the best of our knowledge, this is the first deep learning approach that uses additional nucleus information to improve cell detection and segmentation.
- We present a CNN architecture based on Mask R-CNN and a novel feature pyramid fusion scheme. This CNN architecture shows superior performance in terms of mean average precision compared to early fusion of nucleus and cell signals. It clearly outperforms a state-of-the-art Mask R-CNN [4] applied to cell detection and segmentation with relative mean average precision improvements of up to 23.88% and 23.17%, respectively.

Our approach is related to several instance segmentation approaches for fluorescence microscopy images. Methods that do not rely on deep learning, such as graph cut algorithms [5–9], usually struggle with morphologically complex objects. Similarly, other methods either consider nuclei [5, 10, 11] or segment similarly sized and mostly round objects [12] or different shapes [13, 14]. Most notably, all of these methods consider only a single signal, either cell or nucleus. A method utilizing nucleus information together with the cell signal is described by Held et al. [15] and Wenzel et al. [16]. Their segmentation algorithm uses a fast marching level set [17], i.e., a classic computer vision method that does not rely on machine learning methods. A more recent approach proposed by Al-Kofahi et al. [18] refines deep learning based nucleus segmentation by a seeded watershed algorithm to segment cells.

Recent CNN-based segmentation algorithms can be roughly divided into two groups. Algorithms in the first group perform full image segmentation and require additional post-processing to be applicable to instance segmentation [19–21]. A well-known example in bio-medical image segmentation is the U-Net [22] architecture. However, such methods fail in case of clustered cells. Due to few misclassified pixels, neighboring cells are fused into a single cell, resulting in poor detection performance.

For this reason, we consider methods of the second group of CNN-based segmentation algorithms to be more suitable for our data. These methods perform detection followed by segmentation, i.e., detected bounding boxes are segmented rather than the whole image. For example, van Valen et al. [23] use object detection and perform bounding box segmentation in a subsequent step. Akram et al. [24] describe a CNN architecture using region of interest (RoI) pooling for cell detection and instance-based segmentation. Recently, Mask R-CNN [4] based on Faster R-CNN [25] with feature pyramids [26] achieved state-of-the-art results in natural image object detection and segmentation. Hence, our nucleus and cell detection and segmentation approach is based on Mask R-CNN. However, in contrast to recent work applying Mask R-CNN to a number of segmentation problems in biomedical imaging including nucleus segmentation [27–29], we extend the architecture to meet the requirements of data sets containing both cell and nucleus signals.

## Design and implementation

The fluorescence microscopy images used in our work are collected as part of high-throughput screening to detect treatments that have an influence on the bacterium *Legionella pneumophila* and modify the infection of human macrophages. Microscopic images do not only allow assessment of bacterial replication, but also enable investigations of morphological changes.

### Cell preparation and image acquisition

Monocytic THP-1 cells were obtained from ATCC, inoculated from a −80°C culture and passaged in RPMI-1640 medium with 10% fetal calf serum (Biochrom) at 37°C and 5% CO_2_. The used cell passage numbers were in the range of 5 to 14. Cells were seeded in 100 *μ*l on 96 well Sensoplate Plus plates (Greiner Bio-One) at a concentration of 1.45 × 10^4^ cells per well. Differentiation to macrophages was induced 24 h after seeding by adding 20 nM phorbol 12-myristate 13-acetate (PMA; Sigma-Aldrich) for 24 h. After medium renewal, cells were infected with GFP-expressing *Legionella pneumophila* strain Corby at a multiplicity of infection of 20 for 16 h. For infection, bacteria were plated on BCYE agar, incubated for 3 days at 37°C and 5% CO_2_, resuspended and added to macrophages [30]. After infection, the cells were washed, fixed with 4% paraformaldehyde for 15 min and permeabilized with 0.1% Triton X-100 for 10 min. Staining of cells was achieved with HCS CellMask Red (2 *μ*g/ml; Thermo Fisher Scientific) and Hoechst 33342 (2 *μ*g/ml; Invitrogen) for 30 min.

The images were acquired using an automated Nikon Eclipse Ti-E fluorescence microscope with a 20x lens (Nikon CFI Plan Apo VC 20X) and a Nikon Digital Sight DS-Qi1Mc camera. Finally, the images were converted to TIFF format via the Fiji/ImageJ Bio-Formats plugin.

In total, several hundred thousands of 3-channel images with a size of 1280 × 1024 pixels were collected.

### Dataset of macrophage cells

For our benchmark dataset, 82 representative images were selected and augmented with ground truth information by a biomedical researcher. In our work, only the nucleus and cell channels are of interest.

These 82 2-channel images were randomly split into a training set, containing 64 images, and a test set, containing 18 images. While the training set contains 2044 cells and 2081 nuclei, the test set contains 508 cells and 514 nuclei. The discrepancy between the number of cell and nuclei instances is due to border cells that are not completely visible in the images. Furthermore, some of the cells are in their mitotic phase, i.e., multiple nuclei may occur in a single cell.

Manually generating ground truth labels for segmentation tasks is very time-consuming. Therefore, we generated the ground truth segmentation masks for cell and nuclei instances in a two-step process. In the first step, classical computer vision algorithms were applied to produce an initial segmentation using a threshold-based approach and contour processing functions from the OpenCV library [31] to find cell and nuclei instances. Furthermore, the structures of these instances were analyzed, and cells with more than one nucleus were separated similar to a Voronoi segmentation where each pixel of a cell is assigned to the nearest nucleus. In the second step, these segmentations were manually refined using an image processing tool designed for scientific multidimensional images called Fiji/ImageJ [32]. Most of our manual corrections were necessary at cell to cell borders.

To segment macrophage cells, we follow an instance-based segmentation approach by introducing a new neural network architecture based on Mask R-CNN. It combines nucleus and cell features in a pyramid fusion scheme. The Mask R-CNN [4] architecture is an extension of a Faster R-CNN [25] that predicts bounding boxes with class probabilities and additional instance-based segmentation masks.

Like Faster R-CNN, Mask R-CNN is a two-stage method. In the first stage, a Region Proposal Network (RPN) is used to find object proposals (regions of interest, RoI). Therefore, candidate bounding boxes together with objectness scores are predicted. In the second stage, features are extracted for these candidate bounding boxes using a RoI Align layer that computes fixed-size feature maps through bilinear interpolation. Based on these feature maps, the candidate bounding boxes are refined, classified and segmented using regression, classification, and segmentation heads, respectively. The features of the two stages are shared in a backbone architecture for runtime improvements. The CNN backbone architecture is typically pre-trained on an image classification task. The overall neural network is fine-tuned and trained end-to-end using the multi-task loss *L* = *L*_*box*_ + *L*_*cls*_ + *L*_*mask*_, where *L*_*box*_ is the bounding box regression loss, *L*_*cls*_ is the classification loss, and *L*_*mask*_ is the mask loss (i.e., per pixel sigmoid with binary loss), respectively.

**Fig 2:**
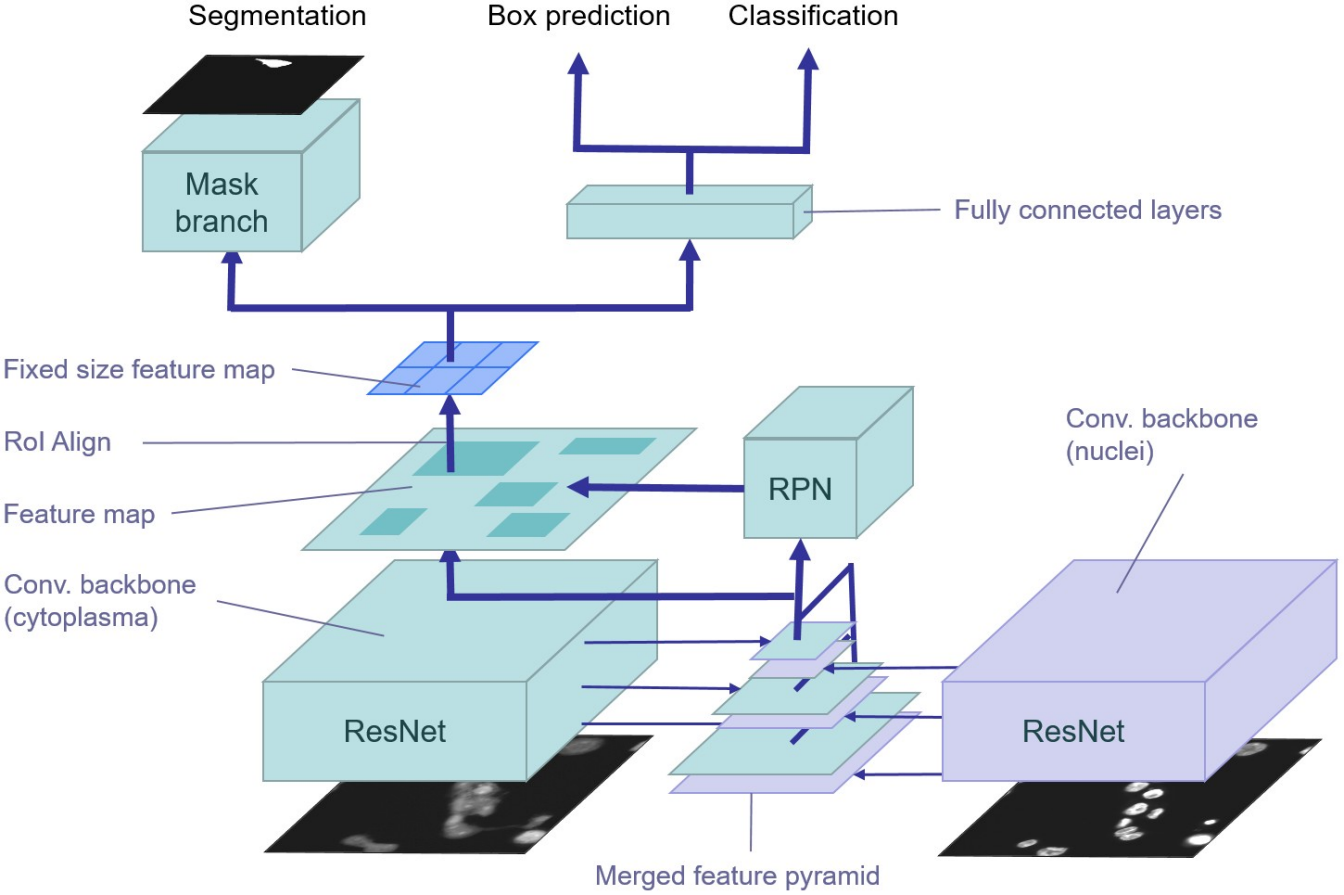
Feature pyramid fusion of nucleus features. Pre-trained nucleus features (violet) are fused with features of the feature pyramid in the cell detection and segmentation model (green) by either concatenation or addition.

To improve detection and segmentation performance within small object regions, Mask R-CNN relies on a Feature Pyramid Network (FPN) [26], a top-down architecture with lateral connections to the backbone layers for building high-level semantic feature maps at all scales. Feature maps for regression, classification, and segmentation of candidate bounding boxes are extracted from the pyramid level according to bounding box sizes. Larger bounding boxes are assigned to higher levels of the pyramid and smaller boxes to lower levels, respectively. In Fig 2, the underlying Mask R-CNN architecture is highlighted in green.

The remainder of this section is organized as follows. We first describe the used backbone CNN. Then, the new network architecture using a pyramid fusion scheme to integrate nucleus features for cell detection and segmentation is presented.

Furthermore, a weighted segmentation loss is introduced to focus the training process on difficult to segment pixels at the cell borders. Finally, we describe the post-processing steps to further improve the detection and segmentation results.

### Reduced ResNet-50 backbone

Residual Neural Networks (ResNet) [33] achieve state-of-the-art performance in natural image classification and object detection tasks. Due to the limited number of object classes (i.e., cells or nuclei), the reduced background noise of fluorescence microscopy images compared to natural images, and the runtime requirements caused by the high-throughput experiments, a reduced ResNet-50 is used as the backbone architecture for our segmentation network. To speed up training, the number of filters as well as the number of building blocks in the ResNet architecture was halved, which results in about ten times less parameters compared to the original ResNet-50. Table 1 shows the reduced ResNet-50 architecture in detail. This architecture was trained for image classification. A pre-trained network for RGB images is not suitable here, since the inputs are 1-channel images for nuclei and cells, respectively. Thus, we converted the ImageNet [34] dataset to grayscale. Based on the 1-channel ImageNet dataset, the backbone model was trained for 120 epochs using batch normalization [35].

**Table 1:** Reduced ResNet-50 architecture.

layer name	output size	blocks
conv1	112 × 112	7 × 7, 64, stride 2
conv2_x	56 × 56	3 × 3 max pool, stride 2 $[\begin{array}{c} 1\times 1,32\\ 3\times 3,32\\ 1\times 1,128 \end{array}]\times 2$
conv3_x	28 × 28	$[\begin{array}{c} 1\times 1,64\\ 3\times 3,64\\ 1\times 1,256 \end{array}]\times 2$
conv4_x	14 × 14	$[\begin{array}{c} 1\times 1,128\\ 3\times 3,128\\ 1\times 1,512 \end{array}]\times 3$
conv5_x	7 × 7	$[\begin{array}{c} 1\times 1,256\\ 3\times 3,256\\ 1\times 1,1024 \end{array}]\times 2$
	1 × 1	avg. pool, fc, softmax

### Feature pyramid fusion

**Fig 3:**
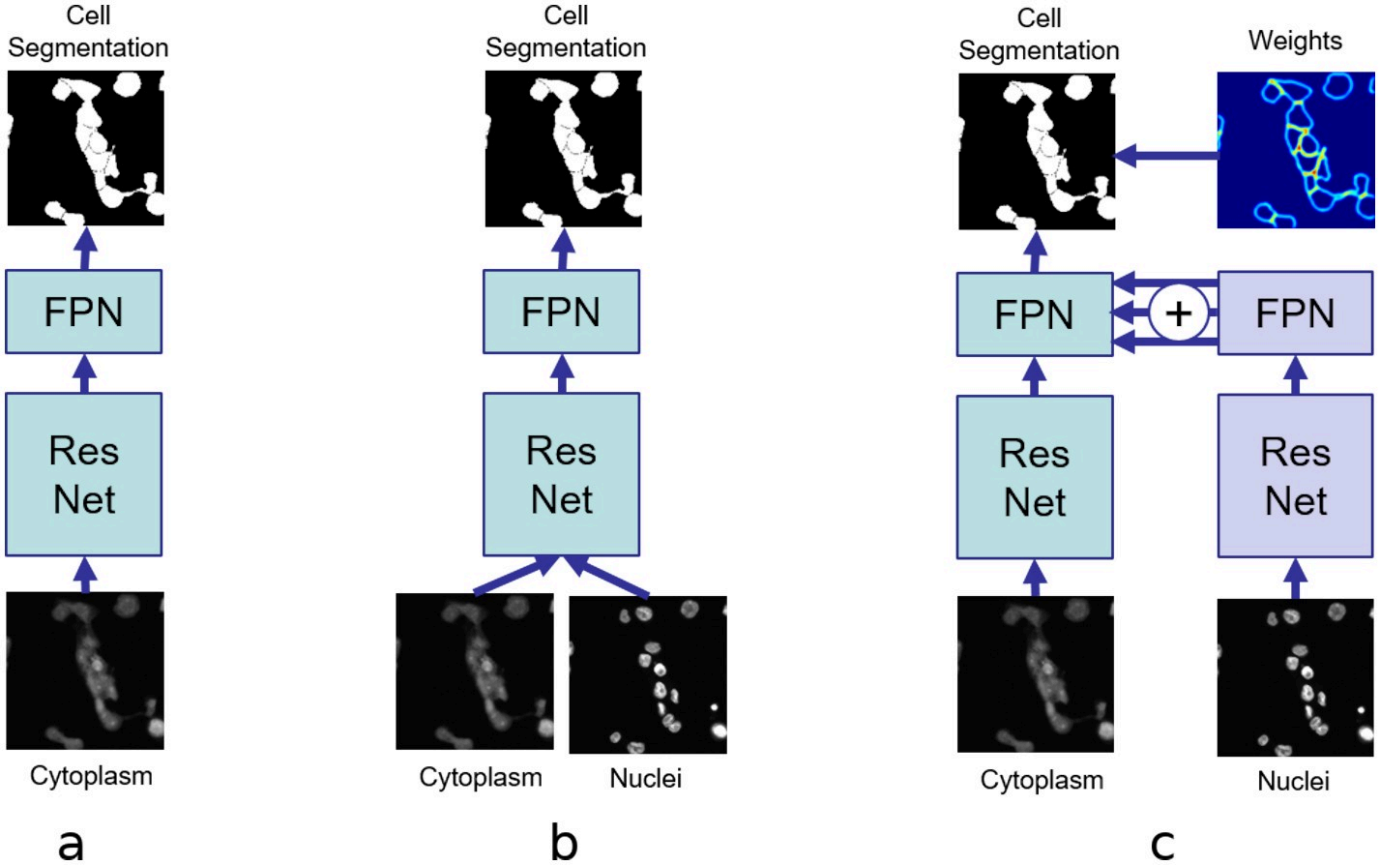
Including nucleus information for cell segmentation. (a) without nucleus information, (b) with additional input for the nucleus channel, and (c) with fused nucleus features.

Fig 3 shows architectures for instance-based cell segmentation using different ways of integrating nucleus information. The architecture in Fig 3a does not include any nucleus information at all. It basically is a Mask R-CNN trained on cell masks for single-class object detection. Similar to an early fusion scheme, the nucleus image is added as an additional channel to the cell image and fed directly into the Mask R-CNN (Fig 3b). However, this architecture cannot utilize available ground truth information for the nucleus channel during the training process.

Nuclei usually have similar shapes and sizes, and in most cases they are clearly separable from each other compared to the appearance of macrophage cells. Therefore, we designed our cell segmentation model in two stages. In the first stage, we train a model for nucleus segmentation to obtain useful nucleus features for the cell segmentation task (Fig 2, violet). This Mask R-CNN architecture using feature pyramids based on the reduced ResNet-50 backbone is trained for nucleus segmentation.

In the second stage, the learned FPN features of the nucleus segmentation task are incorporated into the cell segmentation architecture using feature pyramid fusion (FPF). From each feature pyramid level, we obtain a stack of activations that is merged into the cell segmentation architecture at the corresponding scale. This means that at each stage of the feature pyramid the pre-trained features of the cell nuclei are available and can be used during training in addition to the feature maps for cell segmentation (Fig 2, green). Except for the fused feature pyramids, the backbone architecture for cell segmentation is the same ResNet-50 as for nucleus segmentation.

We also evaluated two merging operations for residual and lateral connections: concatenation and addition. The merged nucleus parameters are fixed during training on cell segmentation, which allows us to combine both models at inference time for concurrently predicting nucleus and cell segmentation masks. The final cell segmentation model has two inputs: the nucleus image and the cytoplasm image. The model architecture was implemented in Tensorflow [36] and is based on a Mask R-CNN implementation [37].

### Weighted segmentation loss

**Fig 4:**
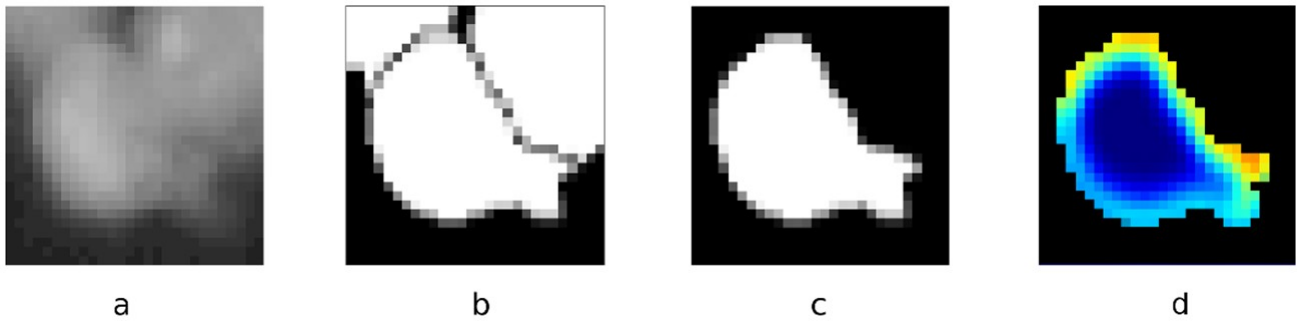
Weights for instances. For each box proposal, crops are resized to 28 × 28 pixels. Crop from the input image (a), full-image segmentation mask (b), cell mask (c) and weight matrix (d).

Since it is more difficult to segment pixels at the cell borders especially within cell clusters, we applied a weighting scheme for the mask loss to focus the model on the edges of cells. The weighting is similar to the weighting used by Ronneberger et al. [22]. By putting more weight to the edges, the model is supposed to learn predicting cell contours more accurately, which requires exact and consistent segmentation masks. We computed a Gaussian blurred weight matrix based on the contours of the cells in the full-image segmentation mask. The Gaussian blur function for pixels *x*, *y* is defined as $G\left(x,y\right)=\frac{1}{2\pi \sigma^{2}}e^{-\frac{x^{2}+y^{2}}{2\sigma^{2}}}$ with the horizontal distance *x* and vertical distance *y* from the origin. We used a 25 × 25 kernel and standard deviation *σ* = 5. Using the weight matrix, pixels near border pixels are weighted up to two times higher than regular pixels, i.e., pixels inside cells. Fig 4(d) shows a visualization of the crop from the weight matrix based on contours computed on Fig 4(b). The crop corresponds to the instance mask in Fig 4(c). It is used for weighting the mask loss for a box proposal processed in the mask branch.

### Post-processing

To further improve the performance of cell detection and segmentation, the following post-processing steps are performed. First, contour processing methods from the OpenCV library are used to detect nucleus regions that are overlapped by more than one cell. In this case, the corresponding cell segmentation masks are merged. Second, a threshold-based segmentation method is applied to find cell regions not covered by the instance-based segmentation masks due to rarely occurring false positives or misaligned bounding boxes. Therefore, the predicted segmentation masks are subtracted from the threshold-based segmentation, morphological operations and contour processing are applied to detect regions, and regions that are smaller than a predefined threshold are discarded. The remaining regions are handled as follows:

- The region is added to a cell if it can be connected to the corresponding cell instance.
- The region is discarded if it is connected to multiple cell instances.
- Otherwise, a new cell instance is generated.

Third, nucleus regions that are not completely covered by cell regions are used to either enlarge the overlapping cell region or to create a new cell with the same shape as the nucleus.

However, in order to ensure a fair comparison, no post-processing was carried out in the experiments when compared to other methods.

## Results

In this section, the performance of the three network architectures shown in Fig 3 is investigated. We additionally compare the performance to the performance of U-Net in order to evaluate how well segmentation without detection (U-Net) performs compared to detection and segmentation with Mask R-CNN architectures. To train the U-Net architecture, we tuned the weighting of the gaps between neighboring cells (or nuclei, respectively) to achieve better results on the corresponding datasets than with the settings used in the original paper [22]. Other training parameters such as learning rate and number of epochs were also optimized to achieve the best results. Since the U-Net model does not return bounding boxes, the contours of cells were used to obtain bounding boxes. In favor of U-Net, contours within contours and very small bounding boxes were ignored in our evaluation. Additionally, we compare the performance to Stardist [12], which achieves state-of-the-art results for nucleus segmentation.

First, the Mask R-CNN architecture in Fig 3a is investigated. Without any information about nuclei, its performance is expected to be lower than for the other architectures.

Second, to verify that incorporating nucleus information is indeed beneficial, Mask R-CNN is extended to accept an additional input channel (Fig 3b). This is a straightforward way of incorporating nucleus information. However, it does not utilize available ground truth segmentation masks for nuclei.

Third, the proposed feature pyramid fusion architecture shown in Fig 3c is evaluated, where pyramid features of a pre-trained nucleus model are combined with cell features using the pyramid fusion scheme. Within this fusion scheme, two merging operations are evaluated: concatenation and addition. Recent work on learning CNN architectures suggests that addition operations are more beneficial in many cases than concatenation operations [38]. Furthermore, the feature pyramid fusion architectures are evaluated in combination with the weighted segmentation loss described above.

The layers of the backbone architecture were initialized with pre-trained weights from an image classification task using the ImageNet dataset. In all experiments, the same pre-trained weights were used for FPF. For the architecture in Fig 3b with two input channels, the pre-trained weights of the first convolutional layer were duplicated and halved to not disturb dependencies to weights of subsequent layers.

In all experiments with FPF, the same training schedule was applied, with a learning rate of 0.001, a momentum of 0.9, and a weight decay factor of 0.0001. The training schedule is as follows: on top of the pre-trained backbone segmentation, regression and classification heads are trained for 100,000 iterations. Next, all layers deeper than conv4 in the backbone are trained for another 250,000 iterations. Finally, the whole network is trained for 500,000 iterations with a reduced learning rate of 10^−4^. Both regression losses and the mask loss are weighted by a factor of 2.

All segmentation models, except the configuration with two input channels, were trained on 512 × 512 1-channel input images. Within the training process, data augmentation was applied to the training images, which increases the number of training images to 497, 355 of size 512 × 512 for each channel, containing 3, 557, 425 nuclei in 4, 288, 104 cells. The variants of the FPF architecture use the same model for nucleus features. It was trained with the same previously described training schedule for cell segmentation models.

All models were evaluated on the test dataset described above. Additionally, to verify that FPF performs better particularly for clustered cells, we created a subset of the test dataset. This subset contains all cell clusters occurring in the test images, but no isolated cells. The resulting 78 images have 256 × 256 pixels. Each image contains at least one cluster of two or more cells. In total, the dataset contains 255 cells with 258 nuclei.

### Evaluation metrics

**Fig 5:**
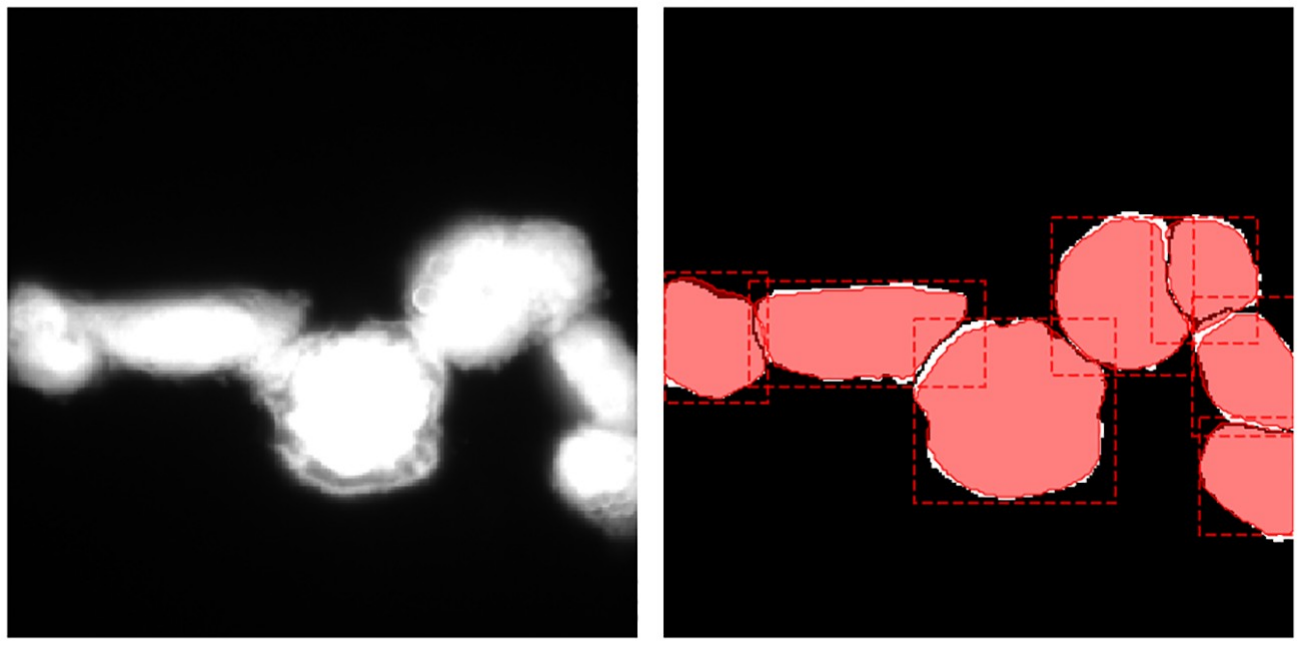
Visualization of cell segmentation errors on a 256 × 256 patch of clustered cells in test data: Predicted masks (red) differ only slightly from ground truth masks (white).

All experiments were evaluated in terms of average precision (AP) for detection and segmentation. AP summarizes the shape of the precision/recall curve. It is defined as the mean precision at a set of equally spaced recall levels. AP is computed for different Intersection over Union (IoU) thresholds. IoU between sets of pixels *A* and *B* is computed as

$$\begin{array}{c} IoU\left(A,B\right)=\frac{A\cap B}{A\cup B} \end{array}$$ (1)

on the 100 top-scoring detections per image for bounding boxes and segmentation masks, respectively. For example, for threshold *t* = 0.5, detections with an IoU overlap of at least 50% with a ground truth object are counted as true positives (TP). Unmatched predicted objects are false positives (FP) and unmatched ground truth objects are false negatives (FN). Precision is computed as $p=\frac{TP}{TP+FP}$ and recall as $r=\frac{TP}{TP+FN}$. We use the 101-point interpolated AP as Lin et al. [39]. AP for a given IoU threshold threshold *t* is computed over all images in the test set as

$$\begin{array}{c} AP\left(t\right)=\frac{1}{101}\sum_{r\in \{0,0.01,...1\}}p_{interp}\left(r\right), \end{array}$$ (2)

where $p_{interp}\left(r\right)=\max_{\tilde{r}:\tilde{r}\geq r}p\left(\tilde{r}\right)$ and $p(\tilde{r}$) is the measured precision at recall $\tilde{r}$. For a detailed description of interpolated AP, we refer to Everingham et al. [40]. The final mean average precision score (mean AP) is the mean over all threshold values between 0.5 and 0.90 with steps of 0.05. However, it should be noted that higher IoU thresholds are less meaningful in our dataset, since in many cases there are no sharp cell borders and thus the corresponding ground truth masks may be somewhat uncertain (see Fig 5).

### Detection and segmentation results

First, we evaluated the performance of the nucleus model used in the FPF architectures (as described in Table 1), Stardist, and U-Net. Table 2 shows results for both detection and segmentation performance in terms of AP on the nuclei test images. Detection and segmentation results are similar, since the majority of the nuclei are round and isolated. However, in some cases nuclei appear adjacent. These instances are hard to separate for U-Net that cannot be trained to detect instances before segmentation. Stardist performs slightly better for segmentation, while Mask-RCNN performs slightly better for detection.

**Table 2:** Detection and segmentation results for the nuclei test dataset in terms of AP.

IoU threshold	0.5	0.55	0.6	0.65	0.7	0.75	0.8	0.85	0.9	mean AP
U-Net *AP*_*det*_	0.674	0.656	0.610	0.604	0.578	0.529	0.418	0.241	-	0.467
Stardist *AP*_*det*_	0.938	0.937	**0.925**	0.909	0.895	0.874	0.852	0.768	0.524	0.847
Mask R-CNN *AP*_*det*_	**0.953**	**0.941**	**0.925**	**0.915**	**0.915**	**0.896**	**0.867**	**0.822**	**0.569**	**0.867**
U-Net *AP*_*seg*_	0.694	0.671	0.634	0.610	0.591	0.528	0.408	0.187	-	0.479
Stardist *AP*_*seg*_	0.942	0.924	0.922	0.905	0.894	**0.892**	**0.880**	**0.812**	**0.600**	**0.863**
Mask R-CNN *AP*_*seg*_	**0.952**	**0.936**	**0.925**	**0.912**	**0.912**	0.890	0.862	0.810	0.536	0.859

Next, the performance of the architecture without any nucleus information, the architecture with two input channels, and the FPF architectures are evaluated for cell detection and segmentation. In the following, ⊙ is used for concatenation and ⊕ for addition. The experiments are conducted on the whole cell test dataset as well as on a subset of the cell test dataset that contains exclusively clustered cells, to have a closer look at those instances that are difficult to detect and segment.

AP scores for detection and segmentation on the cell test dataset are shown in Tables 3 and 4, respectively. As expected, the performance of cell segmentation is much worse when knowledge about nuclei is not incorporated at all. By simply adding one more input channel for the nucleus signal to Mask R-CNN (see Fig 3b), significantly better results are achieved. For the U-Net model, the same method is used to integrate the nucleus channel, i.e., the model has two instead of one input channel. The drawbacks of the U-Net architecture on the cell test dataset are two-fold. First, it cannot use available ground truth of nuclei directly and second, there are even more hard-to-separate signals in this dataset than in the nucleus dataset. Since Stardist is trained for cell segmentation only, it cannot use nucleus information. However, Stardist still performs worse than the Mask R-CNN model without nucleus information. Compared to the performance of Stardist on the nuclei test set, this is most probably caused by the irregular shapes of cells and clustered cells.

**Table 3:** Detection results for the cells test dataset in terms of AP.

IoU threshold	0.5	0.55	0.6	0.65	0.7	0.75	0.8	0.85	0.9	mean AP
U-Net	0.674	0.656	0.610	0.604	0.578	0.529	0.418	0.241	-	0.484
Stardist	0.847	0.814	0.739	0.655	0.566	0.480	0.361	0.247	0.105	0.535
no nucleus	0.886	0.870	0.845	0.790	0.743	0.675	0.548	0.426	0.230	0.668
nucleus input	0.921	0.910	0.886	0.865	0.821	0.727	0.619	0.448	0.221	0.713
FPF ⊙	**0.944**	**0.932**	0.912	0.884	**0.848**	0.771	0.613	0.480	**0.237**	0.736
FPF ⊕	0.935	0.925	0.901	0.886	0.842	0.758	0.633	0.479	0.220	0.731
FPF ⊙ weighted loss	**0.944**	**0.932**	0.897	0.883	0.842	**0.767**	0.633	0.453	0.228	0.731
FPF ⊕ weighted loss	0.942	**0.932**	**0.916**	**0.891**	0.841	0.766	**0.648**	**0.484**	0.224	**0.738**

**Table 4:** Segmentation results for the cells test dataset in terms of AP.

IoU threshold	0.5	0.55	0.6	0.65	0.7	0.75	0.8	0.85	0.9	mean AP
U-Net	0.538	0.471	0.414	0.354	0.295	0.224	0.155	0.077	-	0.283
Stardist	0.869	0.843	0.813	0.773	0.698	0.632	0.504	0.322	0.094	0.616
no nucleus	0.898	0.870	0.858	0.838	0.811	0.751	0.657	0.499	0.226	0.712
nucleus input	0.931	0.909	0.908	0.881	0.866	0.807	0.733	0.585	0.236	0.762
FPF ⊙	0.943	**0.943**	0.920	0.905	0.885	0.830	0.746	0.600	**0.284**	0.784
FPF ⊕	0.935	0.935	0.909	0.908	0.896	0.840	0.762	0.592	0.246	0.780
FPF ⊙ weighted loss	**0.946**	0.934	0.921	0.890	0.876	0.835	0.753	**0.605**	0.253	0.779
FPF ⊕ weighted loss	0.941	0.930	**0.930**	**0.911**	**0.890**	**0.849**	**0.764**	0.603	0.247	**0.785**

FPF performs better than merely using an additional input for Mask R-CNN. However, it does not depend on the merge operation: merging features by concatenation or addition results in similar performance. AP scores for detection (Table 3) and segmentation (Table 4) are similar for all pyramid fusion configurations. Although mean AP performance is slightly better for both architectures trained under a weighted loss, a higher weighting of edges at gaps between cells and near-border pixels in the loss function does not result in a significant performance gain, neither in detection, nor in segmentation. Relative to the mean AP of the model without any nucleus information, the best performing FPF architecture (FPF ⊕ with weighted loss) achieves a performance gain of 10.25%. Compared to the model with the nucleus input channel, the relative performance gain is 3.02%. Similarly, the performance gain for detection is 10.48% (no nucleus) and 3.5% (nucleus input channel). A detailed evaluation including the number of true positives (TP), false positives (FP), and true negatives (FN) of the segmentation results for an IoU threshold of 0.75 is shown in Table 5.

**Table 5:** Detailed cell segmentation results for an IoU threshold of 0.75 on the cells test dataset.

	TP	FP	FN	mean AP
Stardist	379	91	129	0.632
no nucleus	409	93	99	0.751
nucleus input	427	79	81	0.807
FPF ⊙	441	82	67	0.830
FPF ⊕	443	65	65	0.840
FPF ⊙ weighted loss	445	69	63	0.835
FPF ⊕ weighted loss	450	66	58	0.849

**Fig 6:**
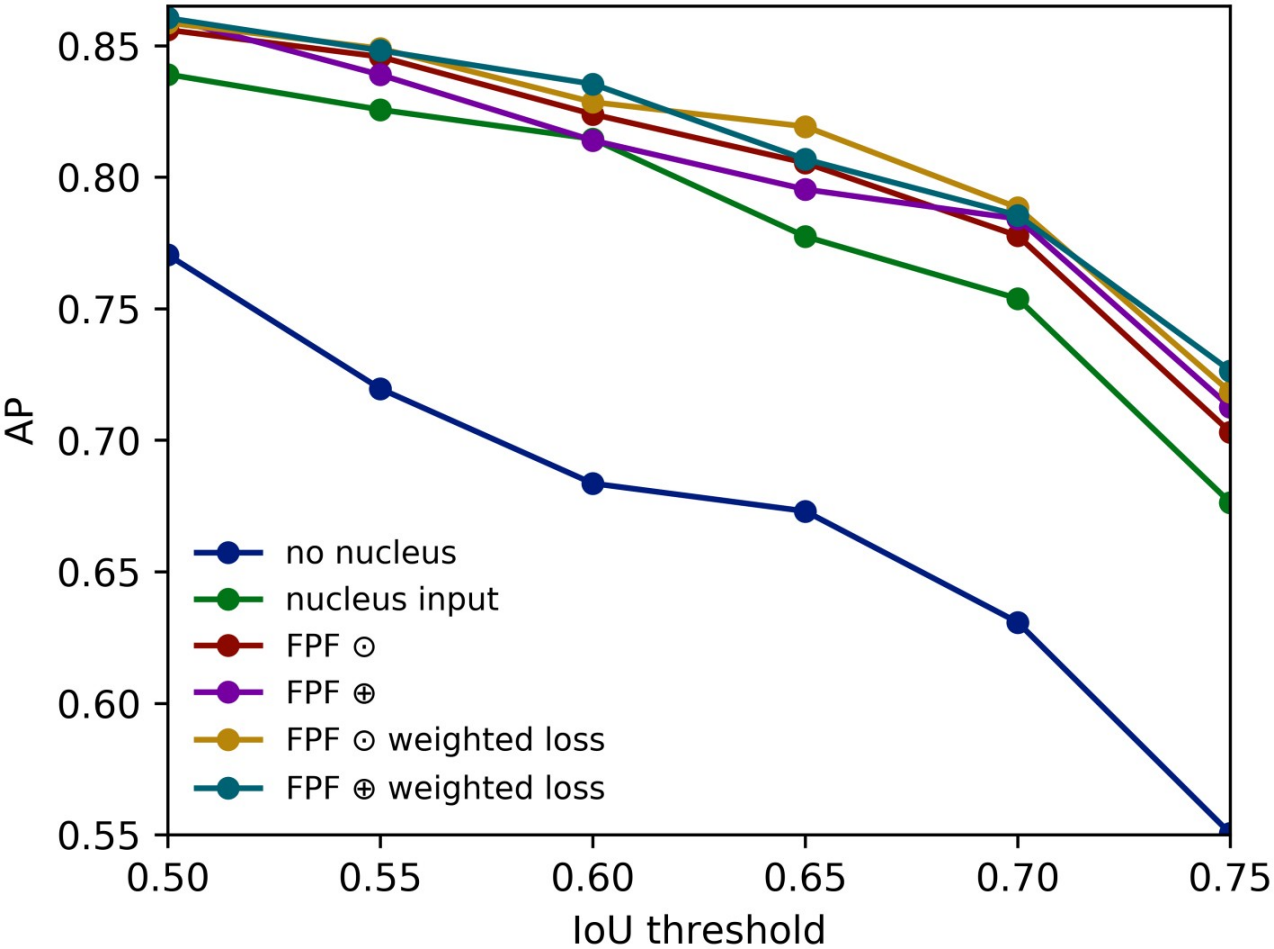
APs for cell segmentation on clustered cells.

**Fig 7:**
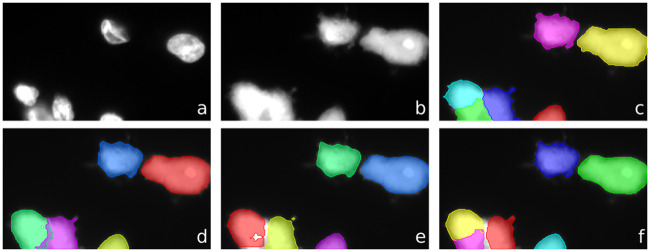
Visualization of segmentation of clustered cells. Top: nucleus signal (a), cytoplasm signal (b), and ground truth segmentation (c). Bottom: Instance segmentations predicted by for model without nucleus information (d), with nucleus channel (e), and FPF ⊕ with weighted loss (f).

The superior performance of the FPF architectures becomes evident when they are evaluated on the clustered cells subset (Tables 6 and 7): a relative performance improvement of 23.88% (no nucleus) and 4.43% (nucleus input channel) for detection in terms of mean AP. Fig 6 visualizes the segmentation results for clustered cells. Fig 7 shows a visualization of the segmentation masks predicted by the model without nucleus information, with nucleus channel, and the best performing FPF architecture.

**Table 6:** Detection results for clustered cells test dataset in terms of AP.

IoU threshold	0.5	0.55	0.6	0.65	0.7	0.75	0.8	0.85	0.9	mean AP
no nucleus	0.772	0.726	0.667	0.623	0.566	0.482	0.334	0.218	0.057	0.494
nucleus input	0.852	0.839	0.792	0.747	0.688	0.581	0.447	0.261	0.066	0.586
FPF ⊙	0.856	0.825	0.825	0.756	0.699	**0.640**	0.438	0.256	0.073	0.596
FPF ⊕	0.851	0.837	0.815	0.763	0.706	0.591	0.473	**0.280**	0.063	0.598
FPF ⊙ weighted loss	0.856	0.847	**0.827**	**0.785**	**0.747**	0.618	0.475	0.271	**0.078**	**0.612**
FPF ⊕ weighted loss	**0.861**	**0.849**	0.825	0.775	0.694	0.625	**0.481**	0.267	0.059	0.604

**Table 7:** Segmentation results for clustered cells test dataset in terms of AP.

IoU threshold	0.5	0.55	0.6	0.65	0.7	0.75	0.8	0.85	0.9	mean AP
no nucleus	0.779	0.720	0.684	0.673	0.631	0.558	0.459	0.319	0.104	0.548
nucleus input	0.839	0.826	0.815	0.777	0.754	0.676	0.589	0.416	0.142	0.648
FPF ⊙	0.856	0.846	0.824	0.805	0.778	0.703	0.615	**0.434**	0.150	0.668
FPF ⊕	**0.860**	0.839	0.814	0.796	0.785	0.712	**0.636**	0.419	0.136	0.666
FPF ⊙ weighted loss	0.859	**0.849**	0.829	**0.819**	**0.788**	0.718	0.625	0.420	0.160	0.674
FPF ⊕ weighted loss	**0.860**	0.848	**0.835**	0.807	0.785	**0.726**	0.619	0.427	**0.161**	**0.675**

**Fig 8:**
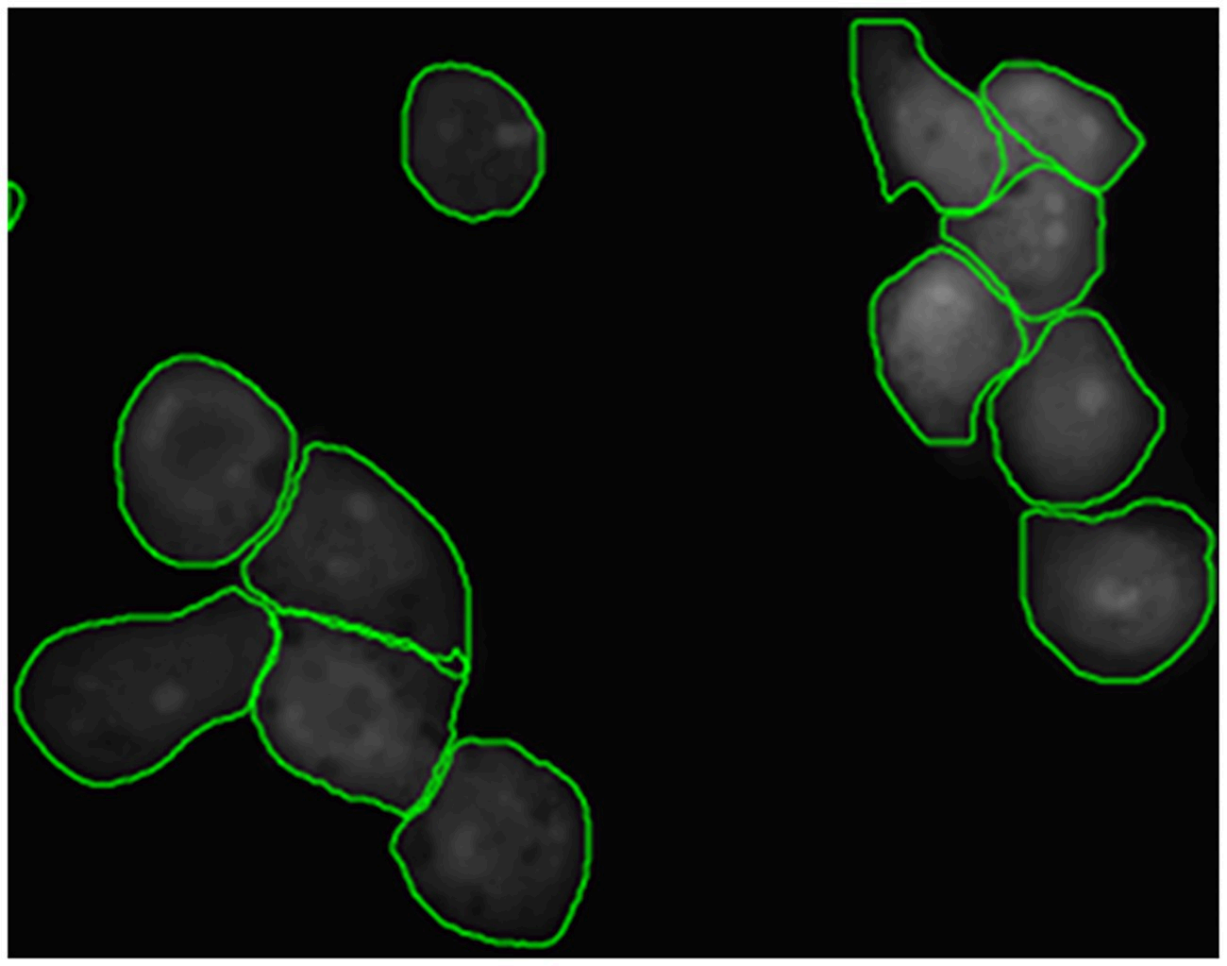
Cell segmentation of clustered cells by Feature Pyramid Fusion (FPF) on a 512 × 512 patch.

Similarly for segmentation, FPF ⊕ with weighted loss performs better. Its relative performance improvement is 23.17% compared to the model without nucleus information, and 4.16% compared to the model with the nucleus input channel, both in terms of mean AP. Fig 8 shows an example of a segmentation performed by FPF ⊕ with weighted loss on a cluster of macrophages. The experiments show that U-Net is not suitable here and the instance-based FPF architectures perform much better. Although Stardist performs well for nucleus segmentation, the performance is considerably lower for cell segmentation.

Using additional post-processing, the detection mean AP on the dataset of clustered cells is improved to 0.65, while the segmentation mean AP increases to 0.682. For the detection mean AP, this is a relative improvement of 31.58% (no nucleus) and 10.92% (nucleus input channel). For the segmentation mean AP, this is a relative improvement of 24.45% (no nucleus) and 5.25% (nucleus input channel).

The runtime of the FPF model is about 222 ms per image using Tensorflow Serving on a server with an Nvidia Geforce GTX 1080 Ti graphics card.

## Availability and future directions

In this paper, we presented a novel deep learning approach for cell detection and segmentation based on fusing previously trained nucleus features on different feature pyramid levels. The proposed feature pyramid fusion architecture clearly outperforms a state-of-the-art Mask R-CNN approach for cell detection and segmentation on our challenging clustered cells dataset with relative mean average precision improvements of up to 23.88% and 23.17%, respectively. Combined with a post-processing step, the results could be further improved to 31.58% for detection and 24.45% for segmentation, respectively.

Code, dataset, and models are publicly available. The dataset is available at https://box.uni-marburg.de/index.php/s/N934NJi7IsvOphf. Code and models are available at https://github.com/umr-ds/feature_pyramid_fusion.

There are several areas for future work. First, sharing backbone weights to build a model that can be trained end-to-end for nucleus/cell detection and segmentation could be interesting. For this purpose, separate Mask R-CNN heads for nuclei and cells have to be attached to the architecture for region proposal generation, regression, classification, and segmentation. Second, the integration of a full-image segmentation loss in addition to an instance-based detection and segmentation loss should be investigated. Third, optimizing the anchor box sampling strategies for nuclei and cells could lead to further performance improvements.
